# Nasal administration of mesenchymal stem cells prevents accelerated age-related tauopathy after chemotherapy in mice

**DOI:** 10.1186/s12979-023-00328-w

**Published:** 2023-01-25

**Authors:** Miriam Zamorano, Jenolyn F. Alexander, Desiree Catania, Shruti Dharmaraj, Annemieke Kavelaars, Cobi J. Heijnen

**Affiliations:** 1 Laboratories of Neuroimmunology, Department of Symptom Research, University of Texas M.D. Anderson Cancer Center, Houston, TX USA; 2 Department of Pediatric Neurosurgery, McGovern Medical School, University of Texas Health Science Center at Houston, Houston, TX USA; 3 Institute of Medical Psychology and Behavioral Immunobiology, University Hospital Essen, University of Duisburg-Essen, Hufelandstr, 55 Essen, Germany

**Keywords:** chemobrain, accelerated aging, cellular therapy, regenerative medicine, cancer treatment, chemotherapy

## Abstract

**Background:**

There is increasing concern that cancer and cancer treatment accelerate aging and the associated cognitive decline. We showed recently that treatment of 9-month-old male mice with cisplatin causes cognitive deficits that are associated with formation of tau deposits in the hippocampus.

Here we explored the capacity of mesenchymal stem cells (MSC) given via the nose to prevent age-related brain tau deposits. Moreover, we more closely examined the cellular distribution of this hallmark of accelerated brain aging in response to treatment of 9-month-old female and male mice with cisplatin.

**Results:**

We show that cisplatin induces tau deposits in the entorhinal cortex and hippocampus in both sexes. The tau deposits colocalize with syndecan-2. Astrocytes surrounding tau deposits have increased glial fibrillary acidic protein glial fibrillary acidic protein (GFAP) expression. Most of the cisplatin-induced tau deposits were located in microtubule associated protein-2 (MAP-2)^+^ neurons that were surrounded by aquaporin 4^+^ (AQP4)^+^ neuron-facing membrane domains of astrocytes. In addition, some tau deposits were detected in the perinuclear region of GFAP^+^ astrocytes and in CD31^+^ endothelial cells. There were no morphological signs of activation of ionized calcium binding adaptor molecule-1^+^ (Iba-1)^+^ microglia and no increases in brain cytokine production.

Nasal administration of MSC at 48 and 96 hours after cisplatin prevented formation of tau deposits and normalized syndecan-2 and GFAP expression. Behaviorally, cisplatin-induced tau cluster formation was associated with reduced executive functioning and working/spatial memory and nasal administration of MSC at 48 and 96 hours after cisplatin prevented these cognitive deficits. Notably, delayed MSC administration (1 month after cisplatin) also prevented tau cluster formation and cognitive deficits, in both sexes.

**Conclusion:**

In summary, nasal administration of MSC to older mice at 2 days or 1 month after completion of cisplatin treatment prevents the accelerated development of tau deposits in entorhinal cortex and hippocampus and the associated cognitive deficits. Since MSC are already in clinical use for many other clinical indications, developing nasal MSC administration for treatment of accelerated brain aging and cognitive deficits in cancer survivors should be feasible and would greatly improve their quality of life.

**Supplementary Information:**

The online version contains supplementary material available at 10.1186/s12979-023-00328-w.

## Highlights

• Cisplatin accelerates formation of tau deposits in entorhinal cortex and hippocampus of older female and male mice.

• Nasal administration of MSC after cisplatin prevents formation of tau deposits in the brain.

• Tau deposits are associated with astrocyte activation but not with activation of microglia.

• Most of the tau deposits in cisplatin-treated mice develop in neurons.

• Nasal administration of MSC reverses cognitive deficits in older mice treated with cisplatin.

**Supplementary Information:**

The online version contains supplementary material available at 10.1186/s12979-023-00328-w.

## Background

Cancer survivors frequently report long-lasting cognitive problems [1–3]. The underlying mechanisms remain to be elucidated, but it is commonly accepted that neurotoxic side effects of chemotherapy and/or radiation play a crucial role [1–3]. The effects of chemotherapy on cognitive outcome in older patients treated for cancer tends to be more severe than in younger patients [4]. However, most preclinical studies on chemotherapy-induced cognitive deficits to date have been performed in young adult rodents. One of the pathways via which cancer and its treatment are thought to lead to cognitive decline is via accelerated brain aging leading to structural abnormalities in gray and white matter [5, 6]. Cancer treatment has shown to increase markers of aging, including reduced telomerase activity, amyloid-β and tau proteins in the peripheral circulation [6–9].

The physiologic function of tau in the brain is to promote polymerization and stabilization of microtubules via binding to the cytoskeletal protein tubulin. This process regulates neurite polarity, axonal sprouting, neuroplasticity, and axonal transport of proteins, mRNAs, and cellular organelles [10, 11]. Changes in the phosphorylation state of tau regulate its normal physiological activities [10, 11]. Post-translational modification of tau including acetylation and hyperphosphorylation and subsequent aggregation can have neurotoxic effects and contribute to cognitive decline in patients with neurodegenerative disorders including Alzheimer disease [10, 11]. Tau pathology in preclinical Alzheimer disease is first observed in the entorhinal cortex and propagates to the hippocampus and neocortex at later disease stages [10, 11]. Chemogenetic attenuation of neuronal activity in the entorhinal cortex reduces tau pathology in the hippocampus in a mouse model of Alzheimer disease showing that tau pathology spreads [12]. Stressors like traumatic brain injury and ischemia can lead to tau phosphorylation and tau deposits as well [13]. Tau deposits also develop in the brain during normal aging, a phenomenon known as primary age-related tauopathy [14]. In normal aging mice, tau deposits are first detectable in the hippocampus at an age of about 12–14 months after which they slowly increase in size and number over time [15]. These age-related tau deposits develop not only in neurons, but also in astrocytes [14]. The latter is known as aging-related tau astrogliopathy (ARTAG) and can occur independently of neuronal tau deposits [16]. Age-related increases in oxidative stress, neuroinflammation and changes in blood brain barrier function have all been suggested to contribute to the formation of tau deposits in the brain.

We showed previously that chemotherapy accelerates the formation of tau deposits in mice. Specifically, we showed that treatment of 9-month-old male mice with cisplatin accelerates the formation of tau deposits in the hippocampus and this phenomenon was associated with cognitive deficits [17]. In contrast, we did not detect these tau deposits in the brain of young (8–10-week-old mice) treated with cisplatin [17, 18]. These previous findings underline the importance of using older mice when investigating the potentially adverse effects of cancer treatment on the brain.

Multipotent mesenchymal stromal cells (MSC) are clinically tested for treatment of diseases and disorders as diverse as graft versus host disease, neurodegenerative diseases, traumatic brain injuries, COVID-19 and cancer [19]. More than 1050 clinical trials investigating the potential of MSC treatment are registered at clinicaltrials.gov. It is well known that MSC can be used across the allogeneic and species barrier. MSC have an immunosuppressive function, which is the rationale for using them to treat graft versus host disease. Therefore, there is no reason to expect problems with rejection. The rationale for using human MSC was that we wanted to know whether these cells eventually would be usable clinically. We already showed that nasal MSC can safely be given to newborns with stroke [20]. Preclinical studies have shown that nasal administration of MSC promote brain tissue regeneration and immunosuppression in models of neurodegenerative diseases and brain injury induced by ischemic stroke or neonatal cerebral hypoxia-ischemia [21–26]. We showed recently that nasal administration of MSC reverses the cognitive deficits induced by treatment of young (8–10-week-old) mice with cisplatin [27, 28]. The nasally administered MSC migrate to the brain parenchyma where they induce a restorative response that results in restoration of structural damage to white and grey matter [27, 28]. However, it is not known whether MSC are also capable of reversing chemotherapy-induced cognitive impairment in older animals.

The aim of the present study was two-fold. First, we performed a careful microscopic study to get more insight into the cellular localization of the tau deposits induced by cisplatin treatment of 9-month-old mice as a sign of accelerated aging. Second, we determined whether nasal administration of MSC after completion of chemotherapy can prevent cisplatin-induced accelerated aging as represented by formation of tau deposits in the brain and the associated cognitive deficits in older male and female mice.

## Results

### Nasal administration of MSC reduces tau deposits in the brain of cisplatin-treated mice

**Fig. 1:**
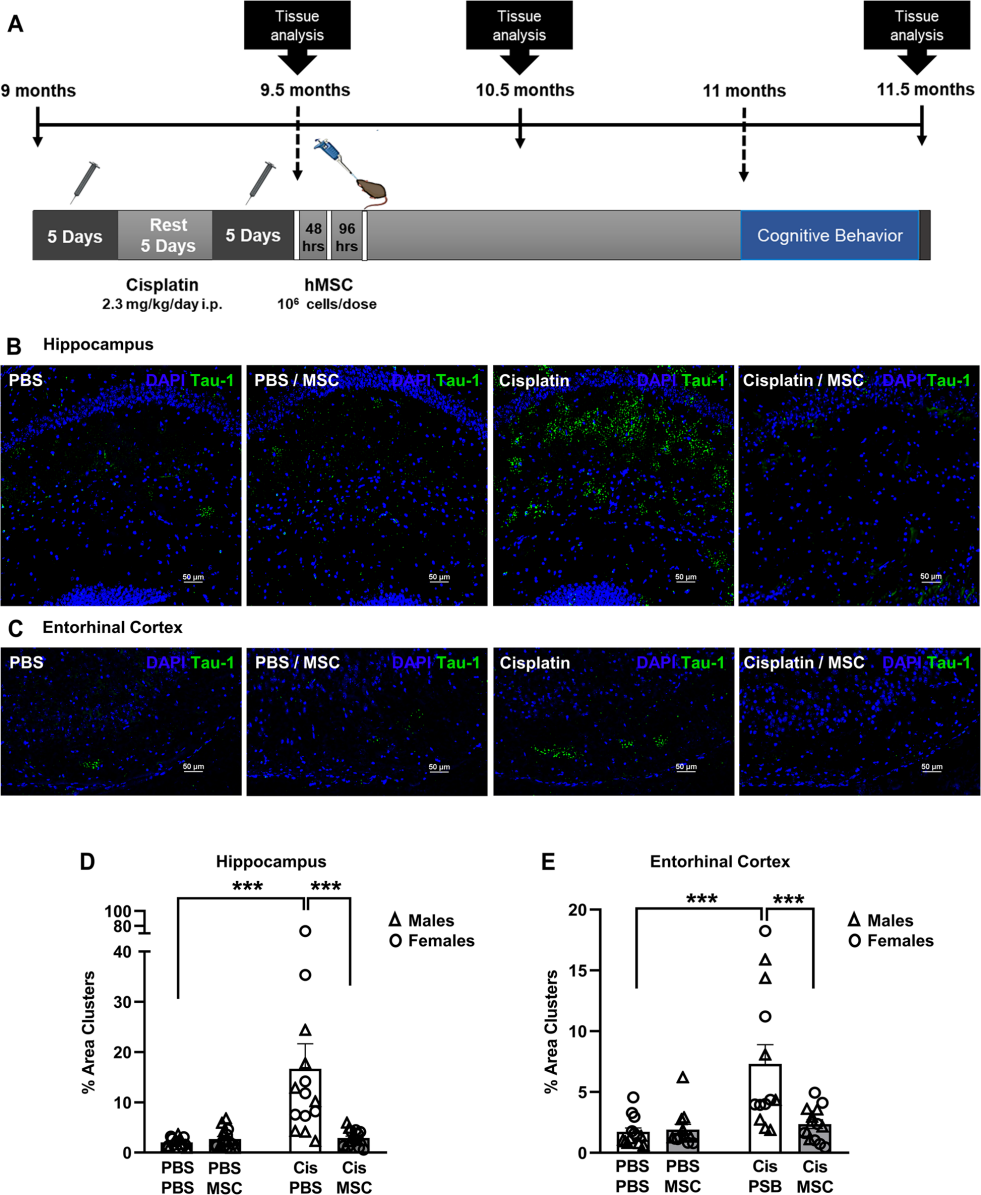
Cisplatin induces tau deposits, and this is reversed by nasal administration of MSC. **A** Experimental timeline. Mice were treated with two rounds of 5 daily doses of cisplatin (Cis; 2.3 mg/kg/day i.p.) with 5 days of rest in between. Mesenchymal stem cells (MSC; 10^6^ cells per dose) were administered intranasally at 48 and 96 hrs after completion of cisplatin treatment. Behavioral assessment was performed 45 days after MSC administration. Brains were collected for immunofluorescence and RT-PCR analysis at the time points indicated. **B** and **C** Representative images of tau immunostaining in coronal hippocampal (**B**) and entorhinal cortex (**C**) sections of 11.5-month-old female mice. (scale bar: 50 μm; Images taken using 20x objective/ 0.75 Numerical Aperture NA). **D** and **E** Quantification of the area of tau deposits in male (triangles) and female (circles) mice expressed as the percentage of total area. *N* = 7 females and 7 males per group. Data are represented as mean ± SEM and were analyzed by Two-way ANOVA with Tukey’s post hoc test. *p < 0.05; **p < 0.01; ***p < 0.001. Three – way ANOVA revealed no statistically significant sex differences.

Treatment of ‘middle-aged’ 9-month old male mice with cisplatin induces signs of tauopathy and cognitive deficits as assessed 2 months later, when the mice are 11.5-months-old [17]. In contrast, we did not detect tau deposits 2 months after cisplatin treatment of young adult (8–10-week-old) mice with cisplatin [18]. Here we expanded our study and investigated whether 9-month-old female mice develop tau deposits in the hippocampus in response to treatment with cisplatin. In addition, we addressed the question whether this sign of chemotherapy-induced accelerated brain aging in male and female mice can be alleviated by cellular therapy. To that end we treated 9-month-old male and female mice with cisplatin followed by two nasal doses of MSC (10^6^ cells/dose) at 48 and 96 hours after the last dose of cisplatin (Fig. 1A). The results in Fig. 1B show that cisplatin also induces the formation of tau deposits in the hippocampus of ‘middle-aged’ female mice as assessed 2.5 months after start of cisplatin treatment when mice have reached the age of 11.5 months. We detected tau deposits in the CA1, CA2, and CA3 region, and in the granular layer of the hippocampus. There were no tau deposits detected in the dentate gyrus (DG).

Further analysis of the brains of cisplatin-treated male and female mice revealed that cisplatin-treated male and female mice also develop tau deposits in the entorhinal cortex (Fig. 1C). We did not detect tau deposits in other parts of the brain, including prefrontal cortex, striatum, amygdala, corpus callosum, paraventricular nuclei, and supraoptic nuclei.

**Fig. 2:**
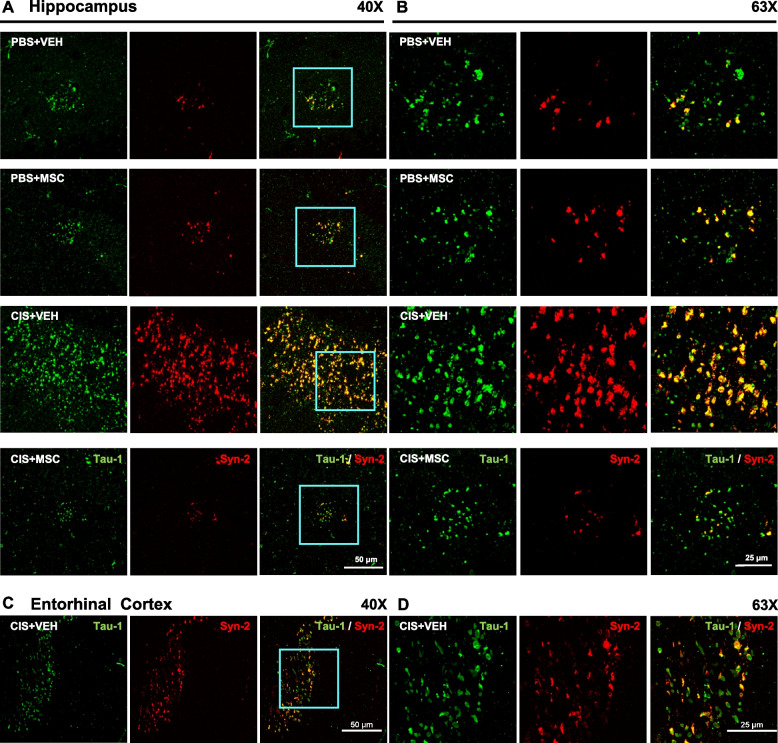
Colocalization of tau deposits with syndecan-2. Brains were collected after completion of behavioral analysis and stained for syndecan-2 (red) and tau (green). Representative images of hippocampus and entorhinal cortex collected with 40X (**A**) and 63X (**B**) objectives show close apposition of tau deposits and syndecan-2 with partial overlap (yellow). Scale bar in (**A**) 50 μm and in (**B**) 25 μm. Syndecan-2 staining reduces along with the reduction in tau clustering in brains from mice treated with MSC after completion of cisplatin treatment.

Nasal administration of MSC at 24 and 48 hours after the last dose of cisplatin decreased the area of tau deposits in the hippocampus to the very low level observed in PBS-treated mice of both sexes (Fig. 1B and D). Nasal administration of MSC also reduced the number of tau deposits in the entorhinal cortex of both sexes (Fig. 1C and E). The tau deposits in the hippocampus and entorhinal cortex co-localized with the heparin sulfate proteoglycan syndecan-2 (Fig. 2), a protein known for its role in cytoskeletal organization, cell migration and its function as an anchor for growth factors and chemokines via its heparin sulfate chains [29]. In models of Alzheimer disease, the syndecan family has been shown to contribute to seeding and spreading of misfolded proteins including tau [29]. Nasal administration of MSC not only normalized tau distribution, but also reduced the deposition of syndecan-2 (Fig. 2).

Kinetic analysis revealed that the cisplatin-induced increase in tau deposits is not yet detectable in the brain of male and female mice immediately after completion treatment (when the mice are 9.5 months old, before dosing the MSC) or at 2 weeks after the last dose of MSC, when the mice are 10 months old (data not shown). These findings indicate that nasal administration of MSC at 48 and 96 hours after completion of cisplatin treatment prevent the fast kinetics of aging as a result of cancer treatment.

### Cellular localization of cisplatin-induced tau deposits

**Fig. 3:**
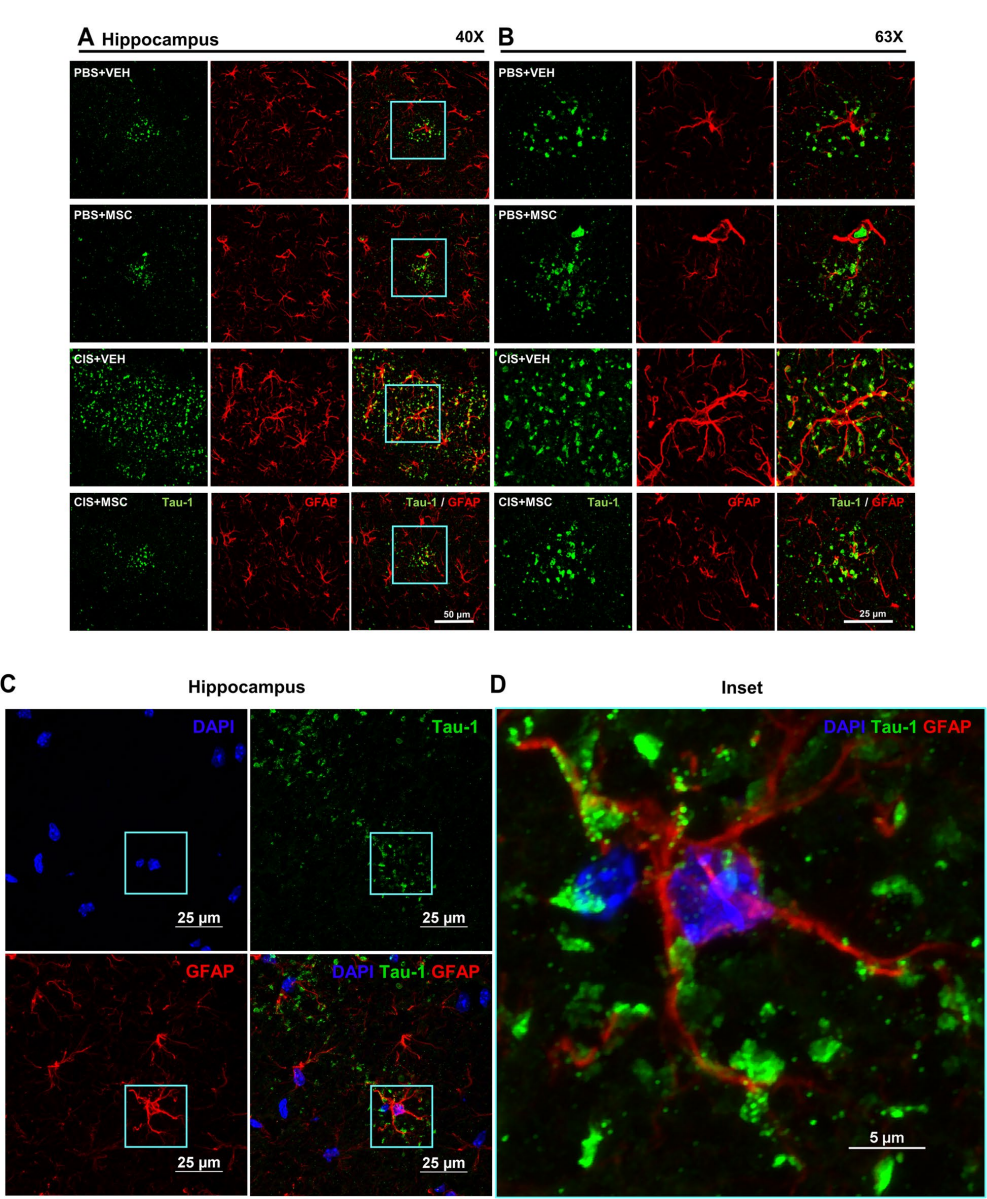
Increased GFAP expression in astrocytes surrounding tau deposits. Representative images of the hippocampus from female mice treated with vehicle or cisplatin followed by nasal administration of MSC with double immunofluorescence labeling of tau-1 (green) and glial fibrillary acidic protein (GFAP; red) in the hippocampus at 40X (**A**) and 63X (**B**). Scale bar in (**A**) 50 μM and in (**B**) 25 μM. **C** High-resolution confocal imaging depicting individual and merged channels of the nuclear marker DAPI (blue), tau-1 (green) and GFAP (red) taken using 100x objective/1.45 NA. Scale bars 25 μm. The region of interest (ROI) in C imaged at higher magnification reveals (**D**) a single GFAP^+^ reactive astrocyte (red) with DAPI^+^ nucleus (blue), with its processes and tau (green) aggregates wrapping each other. Occasionally, tau-1 deposits were detected in the perinuclear region of the astrocyte. Image taken using 100x objective/1.45 NA and 4x scanner zoom. Scale bar 5 μm.

Nasal administration of MSC at 48 and 96 hours after completion of cisplatin treatment prevented the tau cluster formation in the hippocampus and entorhinal cortex of male and female mice as assessed at the age of 11.5-months (Fig. 3A and B). MSC treatment also reversed the increase in GFAP expression surrounding the tau deposits in the hippocampus and entorhinal cortex of cisplatin-treated mice (Fig. 3A and B). In line with our previous findings in male mice [17], we did not detect changes in Iba-1 expression level or distribution in the area surrounding the tau deposits in the hippocampus of cisplatin-treated female mice (Additional file 1 Supplemental Fig. 1). This finding contrasts with what has been reported for patients with Alzheimer disease or its mouse models where there is ample evidence for increased Iba-1 expression [30, 31]. Our low magnification analyses indicated that most of the tau deposits did not colocalize with GFAP. However, high magnification images taken using 100x objective showed that some deposits are closely associated with GFAP^+^ filaments (Fig. 3C and D) or the perinuclear region of GFAP^+^ cells indicating the presence of tau deposits intracellularly in astrocytes (Fig. 3D).

**Fig. 4:**
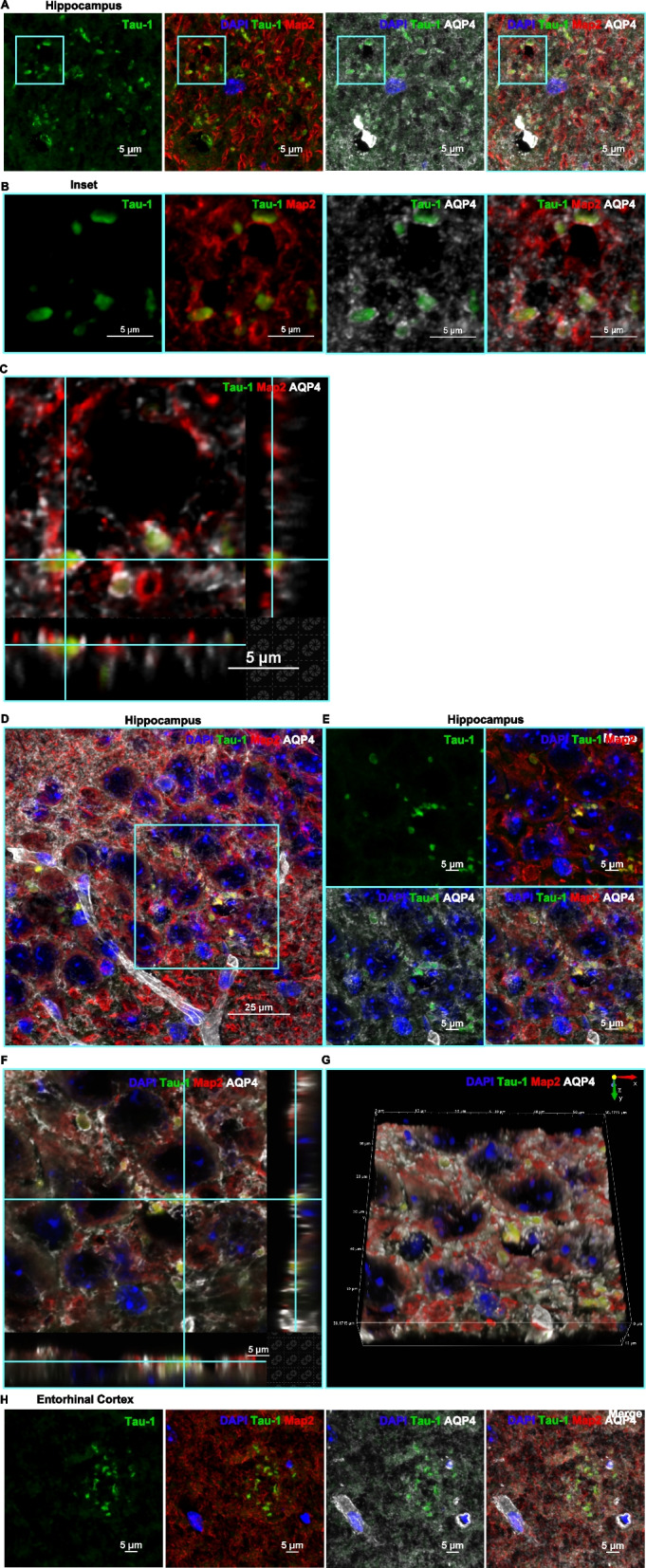
Tau deposits associated with the neuronal marker MAP2. **A** Representative image of the granular layer of the hippocampus showing the presence of tau deposits (green) and their association with the neuronal microtubule associated protein MAP2 (red) and the astrocyte endfeet marker AQP4 (white) with DAPI^+^ nuclei (blue). The region is represented as a single channel of tau-1, 3-channel overlays of DAPI/tau/MAP2 and DAPI/tau/AQP4 and as a 4-channel overlay of DAPI/tau/MAP2/AQP4. Tau (green) and MAP2 (red) colocalization is evident from the yellow signal of the aggregates in the merged or overlaid channels. The multi-channel confocal image was taken using 100x objective/1.45 NA and 2.188x scanner zoom. Scale bar 5 μm. **B** The ROI in A is cropped to show a higher zoom of the tau (green) association with MAP2 (red) and AQP4 (white) expression. The MAP2 neuronal microtubules wrapping the tau-1 aggregates generate the yellow colocalized signal as indicated in the 2- and 3-channel merged images. These neuronal tau aggregates were detected in conjunction with the AQP4-positive disc-like structures which constitute the neuron-facing membrane domains of astrocytes (n-AQP4). **C** Three-dimensional (3D) orthogonal slice view also indicates the tau deposits to be wrapped by neuronal MAP2 and nAQP4 astrocytic domains. Scale bars 5 μm. **D** The pyramidal layer of the hippocampus is showing a similar occurrence of tau deposits (green) and association with neuronal MAP2 (red) and AQP4^+^ astrocytes (white). Image taken using a 100x objective/1.45 NA. Scale bar 25 μm. **E** The region indicated by the ROI in (**D**) captured at a higher magnification and represented as a single, and 3- and 4-channel overlaid images. **F** 3D-orthogonal slice and (**G**) 3D-volume views depicting the occurrence of the tau deposits and their association with neurons and astrocytes. Images taken using a 100x objective/1.45 NA and 2.188 scanner zoom. Scale bar 5 μm. **H** Entorhinal cortex showing the distribution of tau deposits, colocalized with neuronal MAP2 and associated with AQP4. Image taken using a 100x objective/1.45 NA. Scale bar 5 μm.

**Fig. 5:**
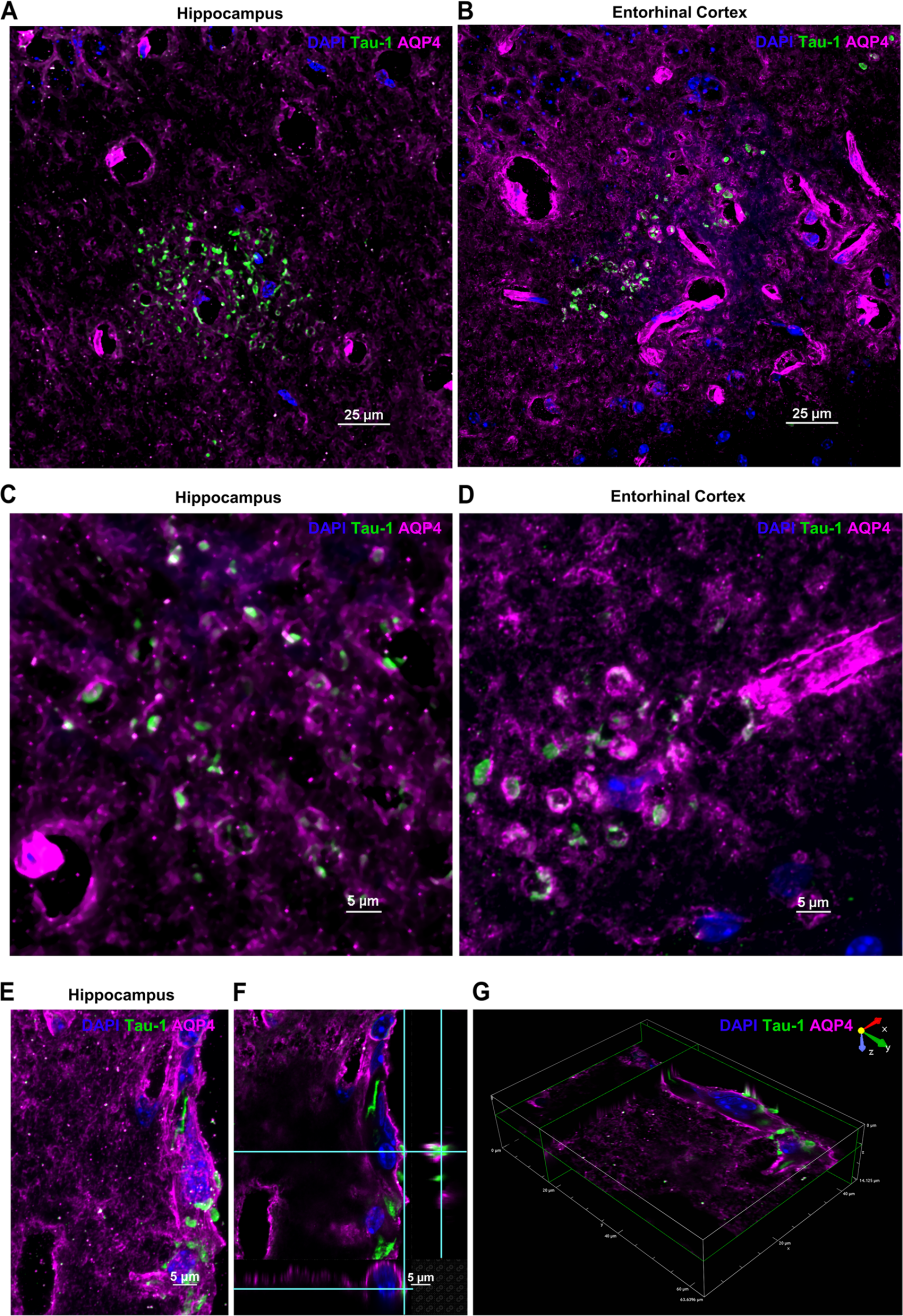
Aquaporin 4 and tau colocalization. **A** Hippocampus and (**B**) entorhinal cortex of cisplatin-treated mouse showing multiple tau deposits (green) and their association with AQP4 (magenta). Images taken using 60x objective/1.4NA. Scale bars 25 μm. The tau deposits frequently occur in the disc-like nAQP4 domains in both (**C**) hippocampus and (**D**) entorhinal cortex. **E **Less frequently, tau deposits were detected in the tubular, perivascular endfeet (p-AQP4) of astrocytes with (**F**) 3D-orthogonal and (**G**) 3D-volume indicating their association with AQP4^+^ astrocytic endfeet and their nuclei. Images taken using 100x objective/1.45NA and 2x scanner zoom. Scale bars 5 μm.

Most of the tau deposits in the hippocampus (Fig. 4A-G) and entorhinal cortex (Fig. 4H) colocalized with MAP2, indicating that they are inside neurons. Indeed, high magnification images reveal wrapping of the deposits by MAP2^+^ microtubules (Fig. 4B-C and E-G). We used aquaporin-4 (AQP4) staining to evaluate the association of tau aggregates with astrocyte endfeet. We observed two patterns of AQP4 staining – channel-like tubular structures representing the perivascular endfeet of astrocytes [32] (p-AQP4) (Fig. 4D, H and 5A-D) and disc-like granular structures indicative of the neuron-facing membrane domains (n-AQP4) of astrocytes [32] (Figs. 4B-C, E-H and 5A-D).

Figure 5

**Fig. 6:**
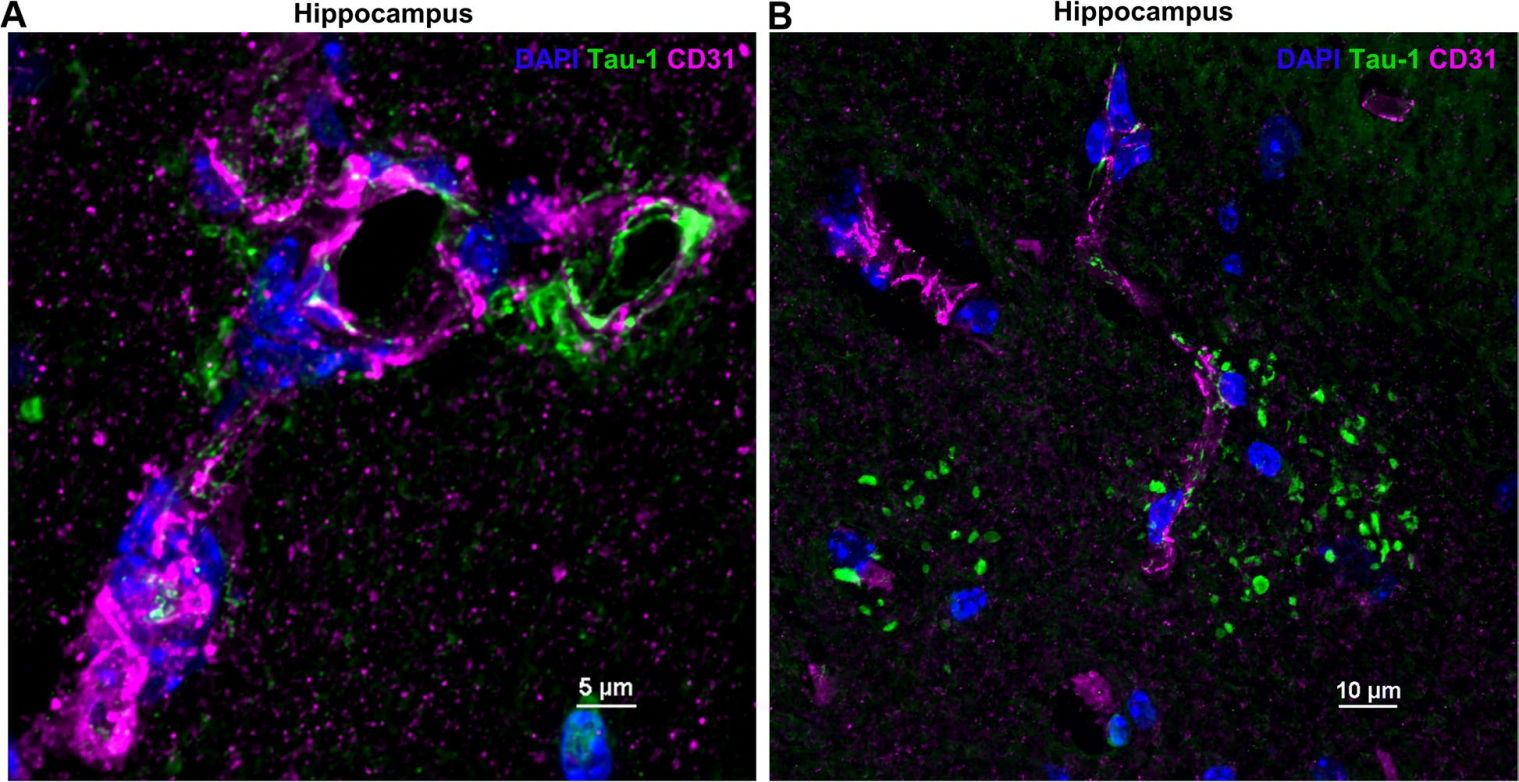
Colocalization of tau-1 deposits with endothelial cells in the hippocampus. Tau deposits (green) associated with CD31^+^ endothelial cells (**A**) (magenta). Occasionally, tau clusters were observed in the (**B**) perinuclear region of endothelial cells as indicated by the colocalized white signal. Images taken using 100x objective/ 1.45 NA. Scale bars 5 and 10 μm.

**Fig. 7:**
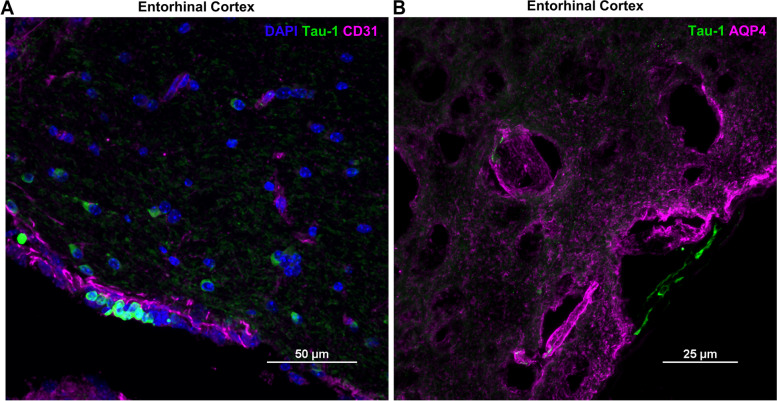
Colocalization of entorhinal cortex tau deposits with endothelial cells and AQP4. **A** CD31^+^ endothelial cells (magenta) and (**B**) AQP4^+^ expression near the entorhinal cortex (magenta) close to the meningeal lining which occasionally contains intracellular tau deposits (green). Images taken using 60x objective/ 1.4 NA and 100x objective/ 1.45 NA. Scale bars 50 and 25 μm.

The tau deposits distinctly appeared at the granular, neuron-facing membrane domains of the astrocytes (Figs. 4B-C, E-H and 5A-D). Three-dimensional (3D) orthogonal slice and volume views reveal that the neurons containing tau deposits (as indicated by the colocalization with MAP2) are wrapped by AQP4^+^ astrocyte endfeet (Fig. 4C, F-G). Some tau deposits also associated with CD31^+^ endothelial cells. (Fig. 6A and B). Interestingly, meningeal cells lining the brain also contained intracellular aggregates of tau (Fig. 7A and B).

### No signs of neuroinflammatory activity or senescence in the brains of cisplatin-treated older mice

We described previously that cognitive impairment in young (8–10-week-old) male and female mice treated with cisplatin was not associated with signs of neuroinflammation as assessed by real-time PCR or RNAseq analysis of hippocampal samples obtained during or at various time points after treatment [18, 27, 33, 34]. In view of the reported increase in neuroinflammation during aging and the localized astrocyte activation in older mice treated with cisplatin, we investigated potential changes in mRNA encoding the prototypic pro-inflammatory cytokines TNF-α, IL-6 and IL-1β in the hippocampus collected 48 hours after completion of cisplatin treatment (at the time of the first dose of MSC when the mice were 9.5 months old) or 2 months after completion of cisplatin treatment and behavioral analysis (11.5 months old). However, also in these older mice no significant changes in these pro-inflammatory cytokines were detected (Additional file 1. Supplemental Fig. 2A-C).

Next we investigated whether cisplatin-induced tauopathy was associated with the expression of senescent markers. We tested *Cdk1na*, *Cdk2na*, and *Hsf1*. We only detected an increase in *Cdkn1a* at completion of cisplatin treatment, when the mice were 9.5 months old. At 2 months after start of treatment, when the mice were 11.5 months old, *Cdnk1a* expression had returned to baseline levels (Additional file 2 Supplemental Fig. 2D). We did not detect expression of *Cdk2na* and expression of *Hsf1* was not changed in response to cisplatin treatment (Additional file 2 Supplemental Fig. 2E).

### Nasal administration of MSC prevents accelerated tau formation and cognitive deficits caused by cisplatin treatment in older mice

**Fig. 8:**
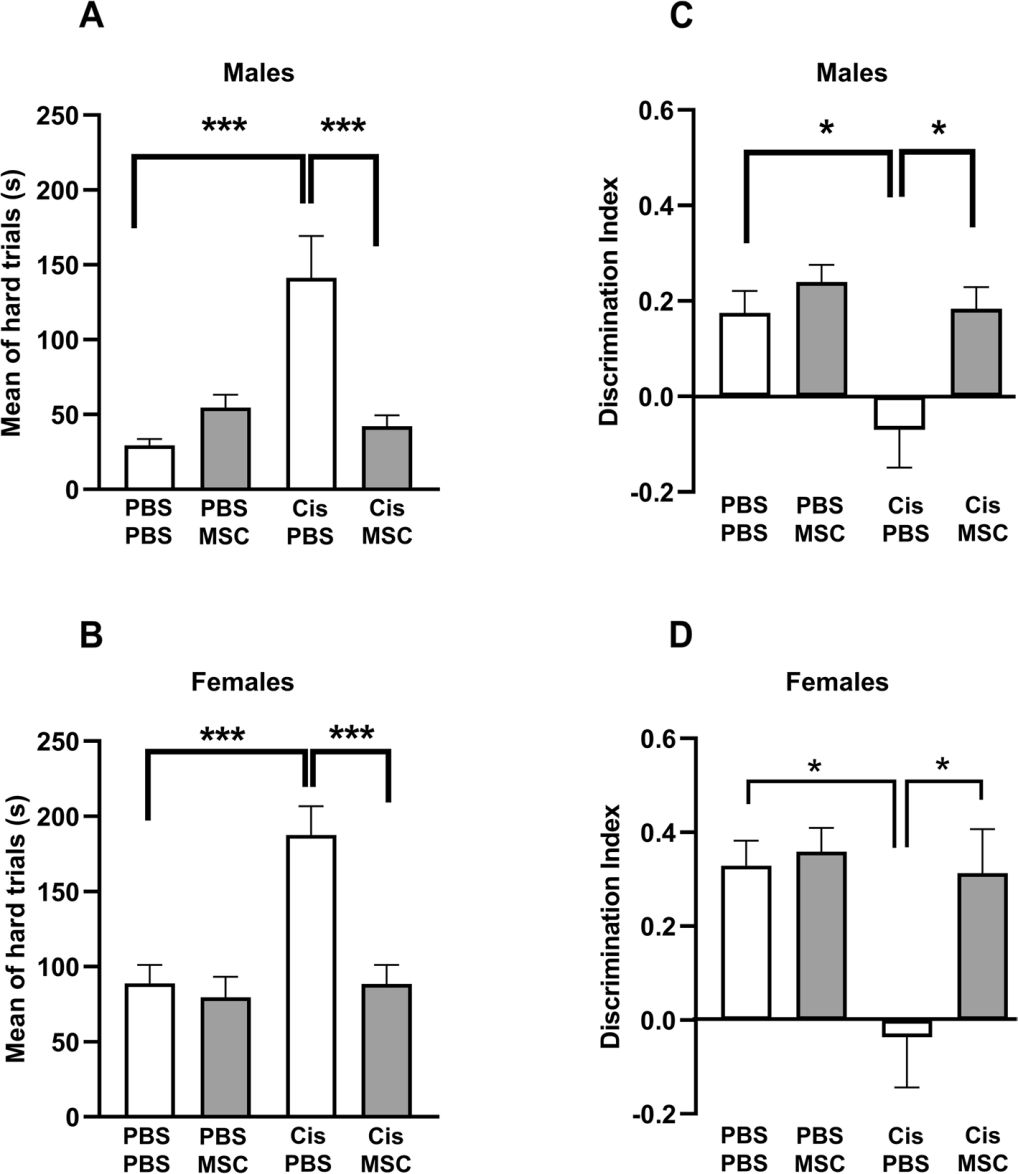
Nasal administration of MSC reverses cognitive impairment induced by cisplatin. Mice were treated with cisplatin and MSC and performance in the puzzle box test (**A** and **B**) and NOPRT (**C** and **D**) was assessed starting 6 weeks after MSC administration. Data for the puzzle box test represent time in seconds needed to enter the dark compartment during the hard trials for males (**A**) and females (**B**). Performance in the NOPRT for males (**C**) and females (**D**) is presented as discrimination index: (Time _Novel_ – Time _Familiar_)/(Time _Novel_ + Time _Familiar_). Results are expressed as mean ± SEM of *n* = 8 mice/group collected in two independent cohorts; ^*^ *p* < 0.05; ^****^p < 0.0001.

We next determined whether the reduction in tau cluster formation after nasal administration of MSC to older mice treated with cisplatin was associated with normalization of performance in behavioral tests for cognitive function. Mice were treated with cisplatin followed by two nasal doses of MSC starting at 48 and 96 hours after completion of cisplatin and behavioral analysis was done 2 months later, when the mice were 11.5 months-old (Experimental set up, Fig. 1A). In the puzzle box test for executive functioning, mice are placed in a brightly lit compartment connected to a dark goal box connected by an underpass. Three sets of trials are performed: easy (open tunnel) Intermediate (bedding in tunnel) and difficult (tunnel blocked with a plug). Male and female mice treated with cisplatin at the age of 9 months and tested when they were 11–11.5 months-old needed more time to enter the dark compartment in the difficult trials (Fig. 8A and B), indicating reduced executive functioning. In line with our previous findings in younger mice, there was no difference in performance in the easy and intermediate task, indicating that the mice were motivated to perform the task (Additional file 3 Supplemental Fig. 3A and B). Notably, nasal administration of MSC at 48 and 96 hours after the last dose of cisplatin normalized performance in the puzzle box test (Fig. 8A and B). Cisplatin treatment also reduced performance in the novel object and place recognition task of older mice (NOPRT). In this test, mice are exposed to two identical objects, placed back in their home cage for 60 minutes and then exposed to one of the now familiar objects and a novel object in a new location. Cisplatin treated male and female mice do not show the preference for the new object in the new location (Fig. 8C and D), indicating impaired memory and spatial orientation. Nasal administration of MSC at 48 and 96 hours after the last dose of cisplatin normalized behavior in the NOPRT. The total time of interacting with the objects did not differ between groups (Additional file 3 Supplemental Fig. 3C and D).

### Delayed administration of MSC prevents tau deposit formation and normalizes cognitive function in cisplatin-treated mice

**Fig. 9:**
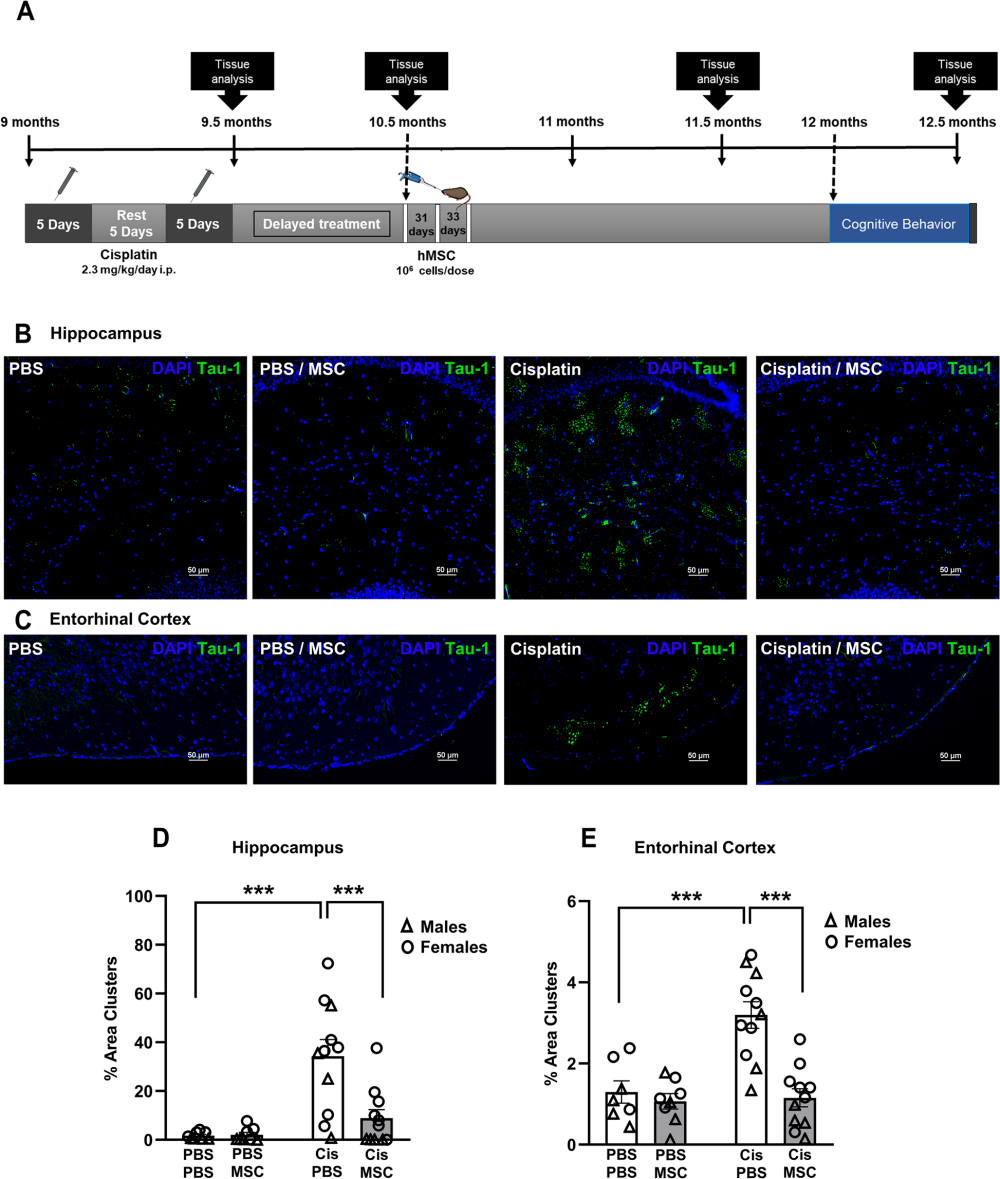
Experimental timeline for delayed treatment with MSC. **A** Male and female mice were treated with 2 rounds of cisplatin for 5 consecutive days and 5 days rest in between. MSC were administered intranasally at 31 and 33 days after cisplatin treatment completion. Cognition assessment was started 6 weeks after MSC administration. Tissue collection was done at the end of the experiment. Representative images of positive reactive immunostaining for tau in hippocampus (**B**) and entorhinal cortex EC (**C**) sections of 12.5-month-old female mice. Images taken using 20x objective/ 0.75 NA. Scale bars 50 μm. Quantification of images from hippocampus (**D**) and entorhinal cortex (**E**) of n = 8 mice (4 male, 4 female) per group. Data are represented as mean ± SEM. *p < 0.05; **p < 0.01; ***p < 0.001 (Two-way ANOVA, Tukey’s post hoc test).

**Fig. 10:**
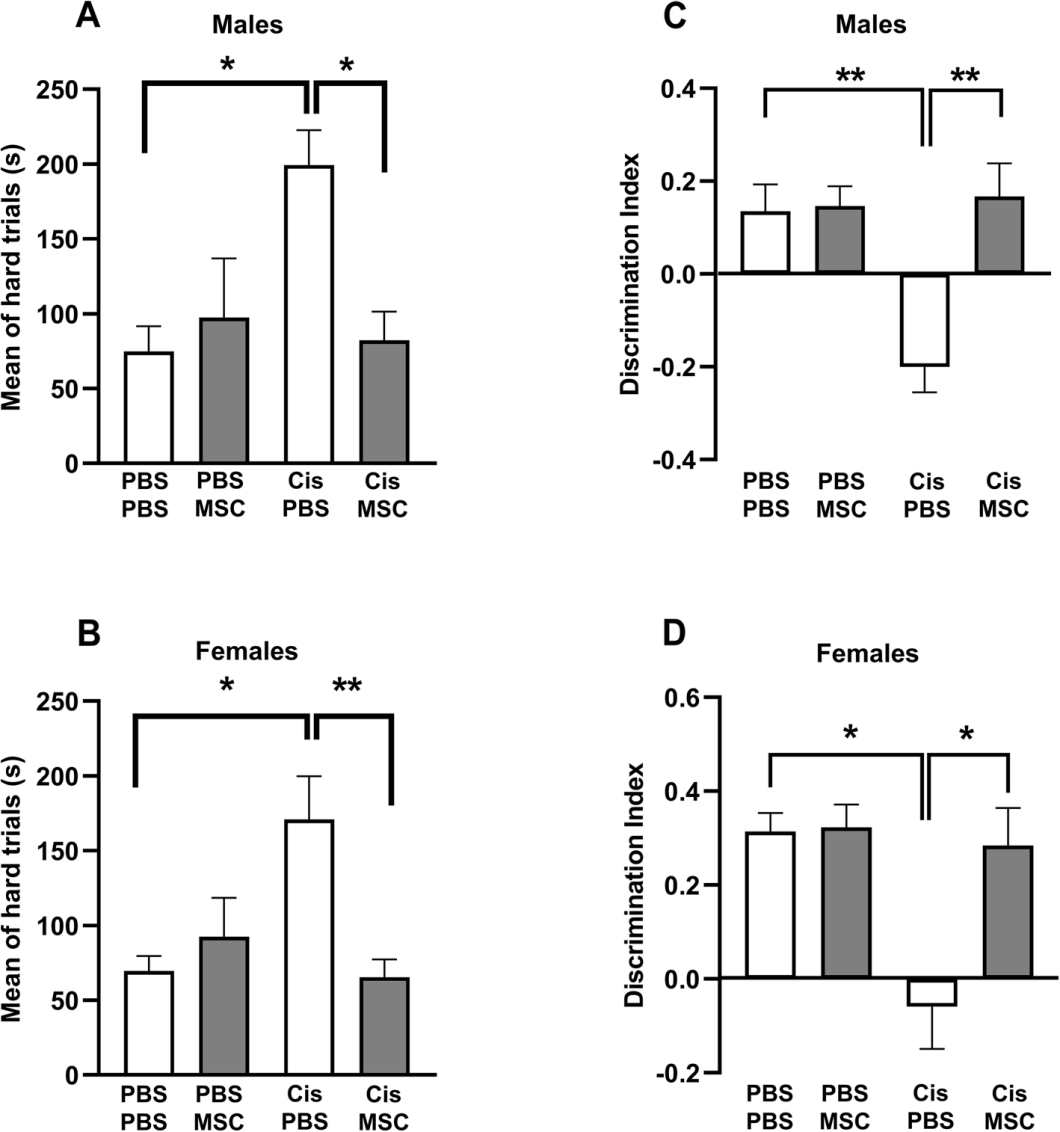
Effect of delayed nasal MSC administration on cisplatin-induced cognitive deficits. Male (**A** and **C**) and female (**B** and **D**) mice were treated with cisplatin followed by MSC and cognitive performance was assessed by PBT (**A** and **B**) and NOPRT (**C** and **D**) as depicted in Fig. 9A. Results are expressed as mean ± SEM; *n* = 8 mice/group (4 males, 4 females); ^*^ *p* < 0.05; ^****^p < 0.0001.

In view of possible clinical translation, we next investigated whether delayed nasal administration of MSC would also be capable of inhibiting the cognitive decline and the tau cluster formation in older mice. To that end, we administered MSC nasally 30 and 32 days after the last dose of cisplatin treatment. Brains were collected at the age of 12.5 months, two months after MSC administration (Fig. 9A). Nasal MSC treatment still reversed tau cluster formation in hippocampus and entorhinal cortex at this late time point (Fig. 9B-E). In both male and female mice delayed nasal treatment with MSC prevented the formation of tau deposits in the hippocampus (Fig. 9D) and entorhinal cortex (Fig. 9E). At 3 months after completion of cisplatin treatment (2 months after MSC administration), performance in the PBT to test executive functioning **(**Fig. 10A and B and Additional file 4 Supplemental Fig. 4A and B) and the NOPRT testing working/spatial memory (Fig. 10C and D) was still impaired in male and female mice treated with cisplatin. We did not detect group differences in total interactions (Additional file 4 Supplemental Fig. 4C and D). These findings indicate that there is no spontaneous recovery of the cisplatin-induced cognitive deficits. Most importantly, delayed treatment with MSC at one month after the last dose of cisplatin normalized behavior in the PBT and the NOPRT in both sexes.

## Discussion

The long-term sequelae of cancer and its treatment include signs of accelerated aging that affect a growing population of cancer survivors [35]. Cognitive decline, a sign of brain aging, is amongst the most devastating of these adverse consequences. Increasing evidence indicates that chemotherapy for cancers outside the nervous system increases the risk of cognitive impairment [3, 5, 6, 36]. Nevertheless, multiple epidemiologic studies have shown that there is an inverse relation between cancer and Alzheimer disease [37–39]. Although this seems counterintuitive, cognitive decline associated with accelerated aging may well be independent of the development of Alzheimer disease. Indeed, a study in patients treated for colorectal cancer showed that chemotherapy increased the risk of cognitive decline, while in the same cohort the risk of developing Alzheimer disease was reduced [40, 41].

Here, we investigated the effect of the chemotherapeutic cisplatin on two signs of accelerated aging; cognitive decline and the formation of tau deposits in the brain. We show that treatment of 9-month-old mature adult male and female mice with cisplatin accelerates the formation of tau deposits in the brain, a hallmark of brain aging. The tau deposits in the brain of cisplatin-treated mice are restricted to the hippocampus and entorhinal cortex. Deposits of tau colocalized with syndecan-2 and were mainly found inside neurons that were surrounded by the aquaporin 4 positive neuron-facing membrane domains of astrocytes. In search for an intervention, we demonstrate that nasal administration of bone marrow derived MSC at 48 and 96 hours after completion of chemotherapy prevented tau cluster formation and normalized cognitive function in male and female mice. Importantly, nasal MSC administration also prevented tau cluster formation and cognitive deficits when treatment was delayed until one month after completion of cisplatin treatment.

In humans, accumulation of tau deposits is thought to be part of normal brain aging and is strongly linked to age-related cognitive decline [14, 42–44]. These tau depositions are first developing in the entorhinal cortex and spread to the hippocampus and other brain areas later in life [44]. Mice also develop tau deposits in the hippocampus during healthy aging and these deposits are first detectable at an age of 12–14 months [15]. Consistently, we detected sparse tau deposits in the hippocampus of control mice at an age of 11–12 months. In addition, we show that there are some tau deposits in the entorhinal cortex of 11–12 months-old mice. Treatment of 9-month-old mice with cisplatin markedly increased the number of tau deposits in both males and females as examined 2 months later. Similar to what has been reported during natural aging, the tau deposits in the brain of cisplatin-treated mice do not label with the AT8 antibody recognizing hyperphosphorylated tau [17]. In contrast, brain AT8 reactivity is increased in mouse models of Alzheimer disease [45]. Moreover, we show that syndecan-2 always colocalizes with the tau deposits in cisplatin-treated mice. No tau^−^ syndecan^+^ clusters were detected. This colocalization is reminiscent of similar protein deposits containing both tau and syndecan-2 in the brain of SAMP8 mice, a well-established model of accelerated aging [46, 47]. Collectively, our current and previous findings indicate that treatment of mature adult mice with cisplatin induces signs of accelerated aging that are prevented by nasal administration of MSC.

Our finding that nasal administration of MSC after completion of cisplatin treatment prevents the formation of tau deposits and the associated cognitive deficits in older mice is especially important from a translational point of view. There are no FDA-approved interventions to manage the cognitive decline that is frequently reported by patients treated for cancer. Currently, most pre-clinical studies aiming at identifying treatments for chemotherapy-induced cognitive decline have been performed in young mice. However, most patients treated for cancer are older adults and aging may affect the efficacy of treatment. It is therefore very encouraging that nasal administration of MSC does not only treat cognitive decline in young [28, 48], but also in older cisplatin-treated mice. Additional clinical relevance lies in our finding that nasal administration of MSC can be delayed for at least one month after the last dose of cisplatin. We showed previously that nasally administered MSC enter the brain of cisplatin-treated young mice where they affect the brain transcriptome, restore synaptosomal mitochondrial function and reverse abnormalities in white matter and synaptic integrity [28, 48].

It remains to be determined how cisplatin treatment accelerates formation of tau deposits in the brain of older mice, but not younger mice and how MSC prevent this tau pathology. Recent studies in show that during healthy aging there is a surge in oxidative stress in brain neurons at an age of approximately 40 weeks (9 months) [49]. Cisplatin treatment is known to induce oxidative stress because it damages neuronal mitochondria [18, 33, 50]. Preventing this mitochondrial damage using a small compound that prevents mitochondrial accumulation of p53, pifithrin-μ, also prevented cisplatin-induced cognitive deficits in young mice [33]. It may well be possible that the cumulative effect of the normal age-related oxidative stress surge and the mitochondrial damage caused by cisplatin causes the formation of tau deposits in the brain of older mice. In support of this model, we showed that nasal administration of MSC reverses the neuronal mitochondrial damage in cisplatin-treated young mice [48]. In vitro, MSC transfer healthy mitochondria to neuronal precursors damaged by cisplatin via actin-based inter cellular structures [51]. This intercellular transfer of mitochondria restores mitochondrial health and neuronal survival [27, 51]. If nasally administered MSC would act similarly in older mice treated with cisplatin, this would alleviate oxidative stress which may well underlie the prevention of the signs of accelerated aging in our current study. We also showed that cisplatin induces peripheral neuropathic pain which is reversed by nasal administration of MSC. We recently published that MSC from IL-10 knockout mice were incapable of reversing neuropathic pain and that intact MSC are capable of inducing IL-10 production in macrophages [51]. Both mitochondrial deficits and IL-10 deficiency can contribute to tau pathology and neuroinflammation [52, 53]. Therefore, we propose that nasal MSC treatment may prevent tau pathology by normalizing brain mitochondrial health and/or IL-10 production.

We show here that the tau deposits are lined by neuron-facing membrane AQP4 in astrocytes. This n-AQP4 has been shown to play a critical role in the clearance abnormal tau from neurons during normal aging via drainage [32]. Notably, aging is associated with a decrease in the capacity of the meningeal lymphatic system to clear damaged proteins [32, 54]. Such reduced clearance may promote the accumulation of tau deposits in the brain and may explain the association of neuronal tau deposits with n-AQP4 of older cisplatin-treated mice, while younger mice may well be capable of clearing the abnormal tau.

Our current finding that cisplatin-induced cognitive deficits are not associated with signs of inflammation in the brain is in line with our previous studies in older female and young male and female mice. Using RNA sequencing, RT-PCR analysis or Iba-1 staining either directly after cisplatin treatment or at different time points after completion of dosing, we did not detect signs of inflammation between control and cisplatin treated mice [17, 34, 48, 50, 55]. These findings contrast with the increased Iba1 staining and brain pro-inflammatory cytokine production in mouse models of Alzheimer disease. It should be noted, however, that the tau pathology in those models is caused by overexpression of human proteins and includes increases in hyperphosphorylated tau as identified using the AT8 antibody.

In conclusion, we show that cisplatin accelerates formation of tau deposits in the hippocampus and entorhinal cortex of older male and female mice. Moreover, we show that nasal administration of MSC up to 1 month after completion of cisplatin treatment prevents this sign of accelerated aging and the associated cognitive deficits.

## Materials and methods

### Animals

Female and male C57BL/6 J mice (Jackson Laboratory) of 9 months of age at start of the study were housed at a temperature of 22 ± 2 °C and 12/12 h reverse dark-light cycle (dark 830–2030 h) with food and water ad lib. Mice were randomly assigned to treatment groups.

### Chemotherapy

Mice were treated with cisplatin in 2 rounds of 5 daily intraperitoneal (i.p.) doses of 2.3 mg/kg in phosphate-buffered saline (PBS) with 5 days of rest in between or with vehicle (PBS) [33].

### MSC

Human MSC were cultured in Minimal Eagle Essential Medium (Corning Life Sciences, Corning, NY, USA) supplemented with GlutaMAX™ (ThermoFisher Scientific, Gibco, Life Technologies Corporation, Grand Island, NY, USA), 2 USP units/mL heparin (Fresenius Kabi USA, Lake Zurich, IL), 7.5% heat inactivated Fetal Bovine Serum (FBS, Sigma-Aldrich, St. Louis, MO, USA), 2.5% platelet lysate (PLTMax®, Mill Creek Life Sciences, Rochester, MN, USA) and 100 units/ml penicillin-streptomycin (Sigma-Aldrich, St. Louis, MO, USA) as described [50]. The cells were positive for CD29, CD44, CD73, CD90, CD105, and CD166. The MSC were and negative for CD14, CD19, CD34, CD45, and HLA-DR. The optimal dose of MSC was previously evaluated in cisplatin-treated younger mice. 0.125 to 1 × 10^6^ MSC were administered per mouse per day at 48 and 96 h after cisplatin treatment. Robust therapeutic effects in the puzzle box test were evident at a dose of 1 × 10^6^ MSC per mouse per day [48]. Thus, we proceeded with this dose for our subsequent studies with nasal administration of MSC. MSC of passage number 3–9 were used in this study. We did not detect any difference in the action of MSC to resolve cognitive dysfunction or decrease tau expression in the brain when using MSC between passage number 3 and 9. On the day of MSC administration, mice first were treated with hyaluronidase in PBS (50 units/3 μL/nostril in per-mouse, Sigma -Aldrich, St Louis, MO). 30 min later, PBS or MSC in PBS were carefully placed in each nostril in a volume of 3 μL, twice applied to each nostril for a total volume of 12 μL containing 10^6^ MSC/mouse [27, 28]. For nasal administration, mice were scruffed by one investigator while a second investigator carefully placed 3 μl doses in one nostril, waited until the fluid was spontaneously inhaled and then applied the next dose in the other nostril with a micropipette.

### Novel object place recognition test (NOPRT)

The novel object-place recognition test (NOPRT) for short term and spatial memory was performed as described [34, 50]. Briefly, each mouse was allowed to explore an arena with two identical objects for 5 minutes and returned to its home cage for 1 hour. During the test phase, one of the now familiar objects was replaced with a different object in a novel location. The time exploring the novel and familiar object during a 5-minute test phase was quantified using EthoVision XT 10.1 video tracking software (Noldus Information Technology., Leesburg, VA) and the discrimination index measure was calculated as (T _Novel_ – T _Familiar_) / (T _Novel_ + T _Familiar_).

### Puzzle box test

The puzzle box test (PBT) for executive function exploits the preference of mice for the dark and was performed as described [27, 34, 50]. Mice are placed in a brightly lit arena (55 cm × 28 cm) connected to a dark area (15 cm × 28 cm) by an underpass (4 cm × 2.5 cm). In the easy trials (trials 1–4), the underpass is open. During intermediate trials (trials 5–7), mice the underpass is blocked by bedding and in the difficult trials (trials 8–11), the underpass is covered by a lid. The time to enter the dark compartment during the difficult trial is recorded as a measure of executive function.

### RT-PCR analysis

Mice were euthanized by CO2 exposure, transcardially perfused with PBS, and the hippocampus was snap frozen in liquid nitrogen. Total RNA was extracted using TRIzol-chloroform, cCDNA was prepared using the high capacity cDNA Reverse Transcription kit (Thermofisher) and quantitative reverse-transcription polymerase chain reaction (qRT-PCR) was performed using PrimeTime qPCR kits for, β-Actin TNFα, CDKn1a, CDKn2a, IL6, IL1β, IL-18 (Integrated DNA Technologies, Coraville, IA, USA). Relative mRNA levels were calculated using the 2-ΔΔCT method and normalized to actin in the same sample. Amplifications without template were included as negative control.

### Immunofluorescence analysis

Mice were perfused transcardially with ice-cold PBS followed by 4% paraformaldehyde (PFA) in PBS. Brains were post-fixed in 4% PFA for 6 hours, cryoprotected in 30% sucrose, frozen in optimal cutting temperature compound (O.C.T., Sakura Finetek, Torrance, CA) and sliced into 9 μm coronal sections. Sections were first blocked in blocking buffer (2% bovine serum albumin (BSA), 5% normal goat serum (NGS) and 0.1% saponin) and then incubated with primary antibodies in antibody buffer (2% BSA, 2% NGS and 0.1% saponin) for 2 hours at room temperature and then for 48 hours at 4C. After which, the sections were washed and incubated with secondary antibodies in antibody buffer for 2 hours at room temperature. The following antibodies were used: mouse anti-Tau-1 (1:200, #MAB3420, MilliporeSigma, Burlington, MA, USA), rabbit anti-syndecan 2 (1:500, #ab191062, Abcam, Cambridge, MA, USA), rabbit anti-GFAP (1:1000, #AP32987SU-N, OriGene, Rockville, MD, USA), chicken anti-MAP2 (1:250, #ab5392, Abcam, Cambridge, MA, USA), rabbit anti-Aquaporin 4(1: 200, AQP-004, Alomone Labs, Jerusalem, Israel), rabbit anti-CD31 (1:25, #ab28364, Abcam, Cambridge, MA, USA), rabbit anti-Iba1 (1:500, #019–19,741, FUJIFILM Wako Chemicals, Richmond, VA, USA), followed by Alexa Fluor 488 goat anti-mouse (1:500, #A11039, ThermoFisher Scientific, Waltham, MA, USA), Alexa Fluor 594 goat anti-mouse (1:500, #A11032, ThermoFisher), Alexa Fluor 594 goat anti-rabbit (1:500, #A11037, ThermoFisher), Alexa Fluor 488 goat anti-chicken (1:500, #A11039, ThermoFisher) or Alexa Fluor 647 goat anti-rabbit (1:500, #A21245, ThermoFisher). Following antibody incubations, the sections were stained with DAPI, washed and mounted using FluorSaveTM (MilliporeSigma, Burlington, MA, USA). To ensure the fluorescent signal is due to the primary antibody and not from tissue background or staining/detection procedure, primary antibody was omitted as a control.

Hippocampal and entorhinal regions were imaged using 20x (0.60 NA), 40x (1.15 NA) and 63x (1.30 NA) objectives with Leica DMI4000 SPE Confocal Microscope (Leica Microsystems GmbH, Wetzlar, Germany) or using 20x (0.75 NA), 40x (1.30 NA), 60x (1.40 NA) and 100x (1.45 NA) objectives with Nikon A1R Confocal Microscope (Nikon Instruments Inc., Melville, NY, USA).

To quantify the tau deposits in the hippocampus and entorhinal cortex, we manually outlined the areas staining positive in 3–4 sections that were cut 100 μm apart per mouse and 4–5 mice per group. The percent area of the hippocampus (also outlined manually) was calculated using ImageJ software [56].

### Statistical analysis

Data are expressed as mean ± SEM and were analyzed by two-way ANOVA followed by Tukey test or using student t-test where appropriate in GraphPad Prism 7.01. For analysis of potential sex differences, three-way ANOVA was performed.

## Supplementary information

**Additional file 1:** **Supplemental Figure 1.** No signs of changes in Iba-1 expression around tau deposits. Brain sections of the mice described in Figure 1 were stained for Iba-1 (magenta) and tau (green) and the hippocampus was imaged. (A and B) PBS-treated and (C and D) cisplatin-treated mice. Images taken using 60x objective/ 1.4 NA. Scale bars 50 μm. The ROIs in A and C imaged at higher magnification (B and D) reveal the ramified morphology of resting microglia. Images taken using 100x objective/ 1.4 NA and 2.198X scan zoom. Scale bar 5 μm.

**Additional file 2:** **Supplemental Figure 2.** Effect of cisplatin on prototypic pro-inflammatory cytokines and senescence markers in the hippocampus. Mice were treated with cisplatin at an age of 9 months as in Figure 1A and mRNA was quantified by RT-PCR in hippocampus collected immediately after completion of cisplatin treatment when the mice were 9.5 months old and after completion of behavioral analysis at an age of 11.5 months. Data were normalized to β-actin and are expressed as mean +/- SEM for *n*= 8 (4 males; 4 females) per group. Two-way ANOVA followed by Tukey test: *p < 0.05.

**Additional file 3:** ** Supplemental Figure 3.** Complete PBT data (A; B) and total interaction time for the NOPRT (C; D) for males (*n*=8) (A; C) and females (*n*=8) (B; D) treated with cisplatin and MSC at the age of 9 months and tested at 11 months as in Figure 8. A, B: Mice were tested in 3 consecutive trials per day for the first 3 days and 2 consecutive trials on day 4. The test is divided into 3 difficulties, easy (day 1 trials 1-3; day 2 trial 4), intermediate (day 2 trials 5-6; day 3 trial 7), and difficult (day 3 trials 8-9; day 4 trial 10). During the easy trials, the animals are allowed free passage between the light and dark compartment via the connecting tunnel. In the intermediate trials, the tunnel is covered with normal bedding and animals must burrow through to access the dark compartment. In the difficult trials, the tunnel is blocked by a lid and the animals must learn to remove the blockage before they can escape to the dark compartment. C, D: Total interaction time in the NOPRT as an index of exploration in males (C) and females (D).

**Additional file 4:** **Supplemental Figure 4.** Effect of delayed treatment with MSC on behavior in the PBT and NOPRT. Complete PBT data (A; B) and total interaction times in the NOPRT (C; D) for males (*n*=4) (A; C) and females (B; D) treated with cisplatin and MSC and tested in the PBT and NOPRT as in Figure 10.

## Data Availability

The datasets generated and/or analyzed during the current study are available from the corresponding author on reasonable request.

## Abbreviations

MSC: mesenchymal stem cells
GFAP: glial fibrillary acidic protein
MAP-2: microtubule associated protein-2
AQP4: aquaporin-4
Iba-1: ionized calcium binding adaptor molecule-1
ARTAG: astrogliopathy
FBS: fetal bovine serum
qRT-PCR: quantitative reverse-transcription polymerase chain reaction
PFA: paraformaldehyde
BSA: bovine serum albumin
NGS: normal goat serum
ANOVA: analysis of variance
