# Supplementary figures and images for: Nasal administration of mesenchymal stem cells prevents accelerated age-related tauopathy after chemotherapy in mice
Source: Immun Ageing. 2023 Jan 25;20:5. doi: 10.1186/s12979-023-00328-w (PMC9874182; doi:10.1186/s12979-023-00328-w)

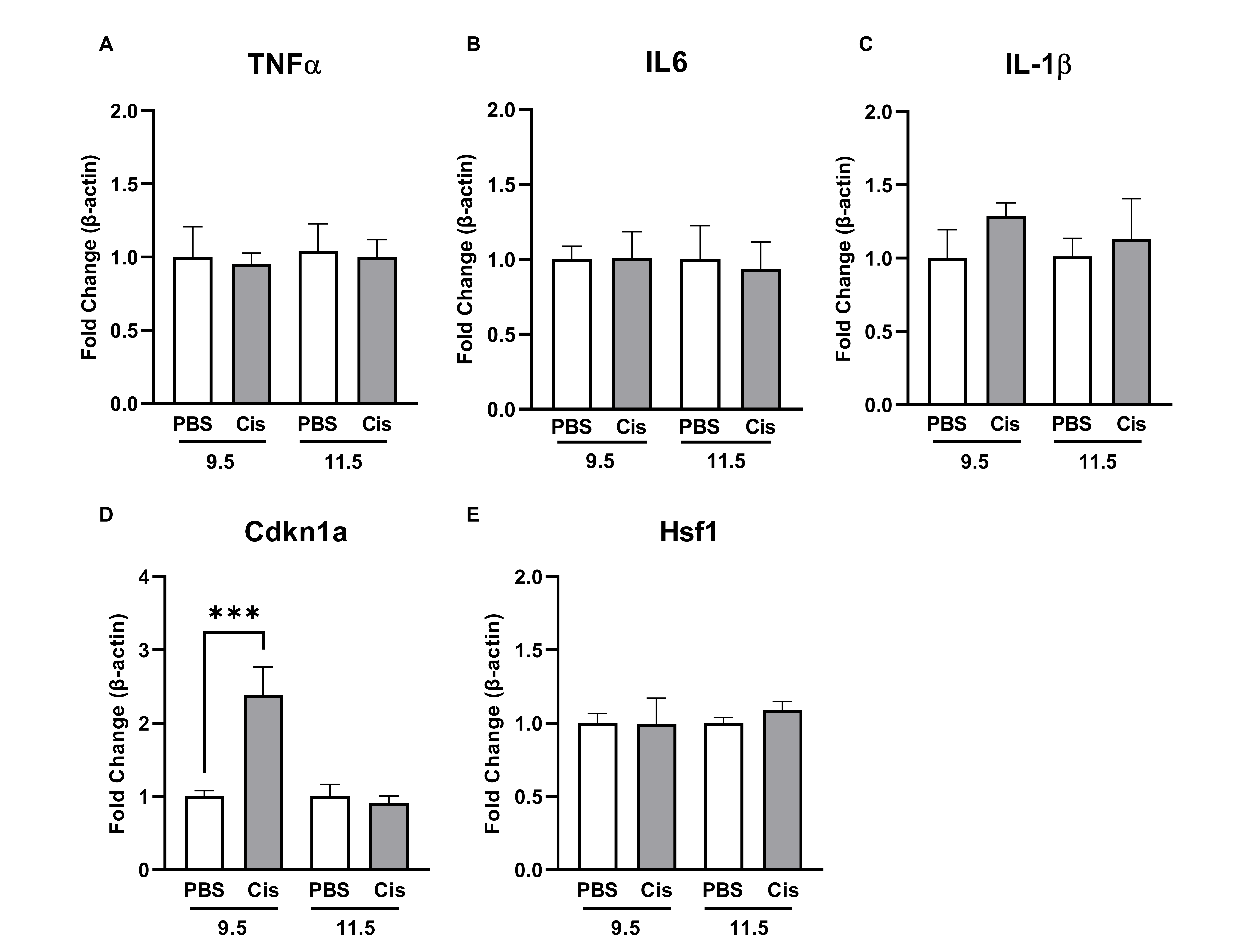

Supplement: Supplementary file 2 — Additional file 2: Supplemental Figure 2. Effect of cisplatin on prototypic pro-inflammatory cytokines and senescence markers in the hippocampus. Mice were treated with cisplatin at an age of 9 months as in Figure 1A and mRNA was quantified by RT-PCR in hippocampus collected immediately after completion of cisplatin treatment when the mice were 9.5 months old and after completion of behavioral analysis at an age of 11.5 months. Data were normalized to β-actin and are expressed as mean +/- SEM for n= 8 (4 males; 4 females) per group. Two-way ANOVA followed by Tukey test: *p < 0.05. [file 12979_2023_328_MOESM2_ESM.tif]
